# Potential Dropout Thoughts and Their Influencing Factors Among Medical Students

**DOI:** 10.7759/cureus.74757

**Published:** 2024-11-29

**Authors:** Sadia Nazir, Attiqa Khalid, Danish Yousaf, Hassan Ali, Muhammad Ahsan Chattha

**Affiliations:** 1 Physiology, Lahore Medical and Dental College, Lahore, PAK

**Keywords:** dropout factors, dropout thoughts, medical education, medical students, mental health

## Abstract

Introduction: Medical student dropout is characterized by the early exit from the medical college prior to graduation. The dropout ratio fluctuates globally and is influenced by factors, such as academic demands, individual characteristics, and insufficient work-life balance, which contribute to thoughts of dropping out. This study sought to evaluate the frequency of dropout ideation and influencing factors among medical students at Lahore Medical and Dental College (LMDC).

Methods: A descriptive cross-sectional study was performed over six months at LMDC, encompassing 459 (n=459) first- to final-year Bachelor of Medicine, Bachelor of Surgery (MBBS) students. A non-probability convenience sampling method was employed. Data were gathered with a standardized questionnaire that encompassed demographic information and enquiries relating to dropout, derived from a previously validated instrument. The analysis was conducted using IBM SPSS Statistics for Windows, Version 26.0 (Released 2019; IBM Corp., Armonk, New York, United States), with descriptive statistics displayed in tables and graphs. A chi-squared test of significance was utilized to examine the relationships between background characteristics and factors linked with dropout thoughts. The threshold for significance is p≤0.05.

Results: Among the 459 students, 174 (37.9%) had thoughts of dropouts, while 50 (28.7%) were seriously contemplating withdrawal. The first year of medical school was considered the most arduous, with 98 (56.3%) students contemplating withdrawal. Eighty-three (47.7%) students indicated that they contemplated dropping out solely on days marked by stress. Notably, social isolation due to medical studies, time management, the impact of a negative event, understanding of medical studies, satisfaction with teaching methodology, experience of homesickness, satisfaction with scores and academic performance, and exam resits all have statistically significant associations with potential dropout thoughts. Furthermore, gender, marital status, transport, and accommodation problems show no significant link with plans to drop out.

Conclusion: The frequency of dropout thoughts among LMDC medical students was markedly elevated. Nonetheless, the contemplation of withdrawal was infrequent. To alleviate the danger of real withdrawal, formal academic advising programs should be established to detect and assist individuals exhibiting early indicators of difficulty.

## Introduction

A medical student dropout refers to an individual who exits a medical school or medical degree program prior to the completion of their studies [1]. The medical profession is demanding and characterized by intense academic and emotional demands, resulting in chronic physical and psychological fatigue. This is encountered by almost all medical students owing to the rigorous demands of medical education. This matter has drawn international interest, leading to multiple and comprehensive studies to investigate the various elements affecting dropout considerations in medical education [2-4]. The choice to abandon a long-held ambition can impose a considerable emotional burden on students, cultivating feelings of sadness, failure, awkwardness, and inadequacy. Elevated dropout rates can damage the reputation of educational institutions and negatively impact perceptions of their support for students and society. Moreover, society may encounter increased pressure on healthcare resources due to a declining number of physicians joining the field. Contemplations over withdrawal from medical school serve as significant indications of future or actual dropout, necessitating meticulous attention from medical educators and policymakers [3].

The dropout rate of medical students in Pakistan is 16%, in China 3.8-26%, and in Saudi Arabia 3.8% [4].

Medical students often correlate dropout thoughts with exhaustion, mental health difficulties, and a perceived inability to manage the rigorous demands of medical education. Factors like an overwhelming academic burden, rigorous educational standards, insufficient time for personal and social engagements, particularly leisure with friends and family, as well as individualized personality attributes such as perfectionism and elevated self-expectations may induce and aggravate thoughts of dropping out. The intention to withdraw from medical school can adversely impact the individual, their family, the faculty, and the broader community [5-8].

It is essential to recognize that students may encounter varying levels of dropout thoughts. Some individuals may experience transient uncertainties during stressful periods, such as examination sessions or upon receiving results that may not align with their expectations [6]. Some individuals may experience more persistent thoughts, leading to a continual struggle that jeopardizes their academic pursuits and increases the chance of prematurely discontinuing their studies. A survey conducted in the United States revealed that roughly 11% of medical students contemplate discontinuing their medical education at some stage throughout their training [7].

A primary element contributing to dropout ideation among medical students is the considerable pressure to achieve academic excellence. The discipline of medicine necessitates that students commit significant study hours, achieve proficiency in a wide range of information, and demonstrate the capacity to apply this knowledge to essential real-world clinical situations. The shift from pre-clinical to clinical training can be intimidating, as the application of this knowledge in clinical contexts may induce heightened anxiety and fear of failure. This worry, combined with performance anxiety, is a significant catalyst for dropout considerations [8]. Moreover, the highly competitive nature of medical study can foster a hostile environment in which students may experience isolation or rivalry. In this context, academically challenged kids may experience embarrassment or hesitance in seeking help, exacerbating and solidifying their thoughts of dropping out [9].

The social and cultural milieu of medical college substantially influences students' considerations of withdrawal. Balancing curricular and co-curricular activities is exceedingly tough; in actuality, it entails prolonged study hours, the sacrifice of personal time, and functioning under significant strain. These may inadvertently cultivate a culture that encourages the notion of withdrawal [10].

A significant element contributing to thoughts of dropping out is financial stress. Students experiencing financial hardships may perceive the supplementary expenditures of hostels, tuition, living expenses, board examinations, and residence applications as burdensome. This financial strain can induce anxiety and emotions of despair, leading to thoughts of withdrawal and students contemplating the cessation of their education [11]. The linkage between financial troubles and the inclination to withdraw from education is well-documented in academic literature, with multiple studies highlighting this relationship among students [12,13]. Diverse life situations, including familial obligations, family disease, personal health challenges, and personal or familial crises, can affect students' lives and contribute to considerations of withdrawing from medical college. Individuals with substantial problems may struggle to balance these responsibilities with rigorous medical education. Gender may influence the experience of dropout ideation. Female medical students may experience distinct pressures, particularly when they perceive a conflict between their personal and familial obligations and the rigorous demands of medical study. This leads to increased stress and consideration of withdrawal [10].

Understanding the underlying motivations and reasons for potential dropout thoughts among medical students is essential for devising effective measures to enhance retention rates and strengthen the healthcare workforce. By understanding the difficulties, educators and policymakers can formulate interventions to address these challenges and cultivate a supportive learning environment that promotes the future and well-being of medical students. The present study sought to evaluate the frequency of dropout ideation among students at a medical college in Lahore and to ascertain the factors contributing to these occurrences.

## Materials and methods

A descriptive, cross-sectional study was undertaken at Lahore Medical and Dental College (LMDC) after obtaining approval from the institute's Institutional Review Board (IRB) and Ethical Committee (EC) (approval number: L-ORIC-08-2024). The study comprised a total of 459 Bachelor of Medicine, Bachelor of Surgery (MBBS) students currently studying in their first to final year. A non-probability convenience sampling method was employed. Data was gathered following the acquisition of informed consent from study participants.

A structured questionnaire served as the instrument for the investigation. The survey comprised two sections. The initial section collected data regarding the sociodemographic data. The second component was a validated questionnaire developed by Abdulghani et al. [7], comprising enquiries on dropout thoughts and associated probable variables. This section comprises questions 1-5 concerning the contemplation of withdrawal and questions 6-15 addressing personal and psychological factors, including previous adverse experiences, social isolation, financial and accommodation issues, and homesickness, while questions 16-19 pertain to academic factors such as teaching methodology challenges, time management, academic performance, and difficulties in comprehending medical studies. Data were entered and analyzed utilizing IBM SPSS Statistics for Windows, Version 26.0 (Released 2019; IBM Corp., Armonk, New York, United States). Data was exhibited in the form of tables and graphs. Descriptive statistics were employed utilizing numerical values and percentages. A chi-squared test of significance was utilized to examine the relationships between background characteristics and factors linked with dropout thoughts. The threshold for significance is p≤0.05.

## Results

A total of 459 MBBS students, from first to final year, participated in the study (n=459).

Sociodemographic background of students and considerations about dropout

Among the participants, 189 (41%) were men and 270 (58%) were women. A predominant 442 (96.3%) of these students were single, with 251 (54.7%) attending as day scholars. Approximately 79 (17%) students said that one or both of their parents are physicians, while 88 (20%) had siblings who are doctors. Notably, none of the sociodemographic factors of the participating students indicate a significant association with dropout intentions (Table 1).

**Table 1:** The relation between sociodemographic variables and dropout thoughts. FSC: Faculty of Sciences

Variables	Categories	Frequency (%)	Thoughts of dropping out from medical school		x^2 ^(p-value)
			Yes	No	
Gender	Male	189 (41)	75 (16.3)	114 (24.8)	0.43 (0.512)
	Female	270 (58)	99 (21.5)	171 (37.2)	
Marital status	Married	17 (3.7)	6 (1.3)	11 (2.4)	0.05 (0.821)
	Unmarried	442 (96.3)	168 (36.6)	274 (59.7)	
Residential status	Day scholar	251 (54.7)	98 (21.4)	153 (33.3)	0.30 (0.858)
	Boarder	181 (39.4)	66 (14.4)	115 (25)	
	Other	27 (5.9)	10 (2.2)	17 (3.7)	
High school qualification	FSC	376 (81.9)	138 (30)	238 (51.9)	1.90 (0.386)
	A- Level	76 (16.6)	32 (6.8)	44 (9.6)	
	American Board	7 (1.5)	4 (0.9)	3 (0.6)	
Doctors in the family	Father	57 (12.4)	26 (5.6)	31 (6.7)	2.33 (0.674)
	Mother	22 (4.8)	7 (1.5)	15 (3.3)	
	Brother	37 (8.1)	14 (3)	23 (5)	
	Sister	51 (11.1)	21 (4.6)	30 (6.5)	
	None	292 (63.6)	106 (23.1)	186 (40.5)	

Student thoughts of dropping out from medical school

Table 2 indicates that 174 (37.9%) of the students contemplated withdrawing from medical school. Among these, only 25 (14.4%) students consistently contemplated withdrawing from medical school. Nonetheless, the majority (83, 47.7%) of medical students contemplated withdrawal alone on particularly stressful days. Encouragingly, the percentage of students who seriously considered dropping out was merely 50 (28.7%). The first year of medical school seemed to be among the most challenging phases of medical education, with a significant proportion (98, 56.3%) of students contemplating leaving.

**Table 2:** Student thoughts of dropping out from medical school.

Variables	Categories	Frequency (%)
Have you ever thought of dropping out from medical college?	Yes	174 (37.9)
	No	285 (62.1)
If yes, then in which academic year did you think of dropping out from medical college?	First	98 (56.3)
	Second	49 (28.2)
	Third	16 (9.2)
	Fourth	7 (4)
	Fifth	4 (2.3)
How often do you think of dropping out from medical college?	Always	25 (14.4)
	Often	30 (17.2)
	Sometimes	67 (38.5)
	Rarely	52 (29.9)
When do you think of dropping out from medical college?	Throughout the year	53 (30.5)
	On a specific day	83 (47.7)
	In a specific academic year	38 (21.8)
How seriously do you think of dropping out from medical college?	Very seriously	50 (28.7)
	Not seriously	124 (71.3)

Non-academic variables and dropout thought

Table 3 illustrates the non-academic characteristics and their associated dropout considerations. Eighty-one (17.6%) students experienced adverse occurrences in the last year, the inclination to withdraw (p=0.03). Furthermore, a diminished sense of personal achievement and social isolation resulting from medical studies was associated with heightened dropout intentions; 126 (27.5%) students who felt socially isolated had dropout intentions (p=0.00). Ninety-five (27.8%) students who experienced homesickness were more likely to contemplate dropping out compared to those who were not (p=0.02).

**Table 3:** Non-academic variables and dropout thought. *: p-value is statistically significant

Variables	Categories	Frequency (%)	Thoughts of dropping out from medical school (frequency)		x^2^ (p-value)
			Yes	No	
Are you currently facing any financial problems?	Yes	44 (9.6)	22 (4.8)	22 (4.8)	3.02 (0.08)
	No	415 (90.4)	152 (33.1)	263 (57.3)	
Do you feel medical study has isolated you?	Yes	278 (60.6)	126 (27.5)	152 (33.1)	16.46 (0.00*)
	No	181 (39.4)	48 (10.5)	133 (28.9)	
Have you experienced any negative event that had an impact on your studies in recent times (major personal illness and a major illness in a close/family member)?	Yes	185 (40.3)	81 (17.6)	104 (22.6)	4.54 (0.03*)
	No	274 (59.7)	93 (20.3)	181 (39.4)	
Have you faced any difficulty in understanding medical studies?	Yes	258 (56.2)	113 (24.6)	145 (31.6)	8.68 (0.00*)
	No	201 (43.8)	61 (13.2)	140 (30.5)	
Are you satisfied with the methodology of teaching?	Yes	212 (46.2)	68 (14.8)	144 (31.3)	5.69 (0.01*)
	No	247 (58.8)	106 (23)	141 (30.7)	
Who decided that you entered into medical school?	Oneself	312 (68)	106 (23)	206 (44.8)	6.40 (0.01*)
	Parents	147 (32)	68 (14.8)	79 (17.2)	
Do you face any transportation problems?	Yes	105 (22.9)	48 (10.5)	57 (12.4)	3.52 (0.06)
	No	354 (77.1)	126 (27.5)	228 (49.7)	
If you stay in a hostel, are you satisfied with the accommodation?	Yes	94 (30)	32 (10.2)	62 (19.8)	1.74 (0.18)
	No	219 (70)	92 (29.4)	127 (40.6)	
If not living at home, do you experience homesickness?	Yes	222 (64.9)	95 (27.8)	127 (37)	5.39 (0.02*)
	No	120 (35.1)	36 (10.5)	84 (24.5)	

Academic variables and dropout thought

In Table 4, 110 (23.9%) medical students who expressed dissatisfaction with their score percentage and academic performance contemplated withdrawal (p=0.03). Eighty-five (18.5%) students with an average score percentage of 70-89% over the previous academic year were classified as dropout thinkers (p=0.00). Similarly, 39 (8.5%) students who had repeated an academic year throughout their medical training had thoughts of dropping out (p=0.02). One hundred and thirty-eight (30%) students who contemplated withdrawal also encountered time management issues during their studies (p=0.00).

**Table 4:** Academic variables and dropout thought. *: p-value is statistically significant

Variables	Categories	Frequency (%)	Thoughts of dropping out from medical school (frequency)		x^2^ (p-value)
			Yes	No	
Are you satisfied with your score percentage and academic performance?	Yes	198 (43)	64 (13.9)	134 (29.1)	4.615 (0.03*)
	No	261 (56.9)	110 (23.9)	151 (32.9)	
What was your average score percentage during the last academic year?	90% or above	50 (10.9)	18 (3.9)	32 (6.9)	15.82 (0.00*)
	70-89%	262 (57.1)	85 (18.5)	177 (38.5)	
	50-69%	136 (29.6)	62 (13.5)	74 (16.1)	
	Below 50%	11 (2.4)	9 (1.9)	2 (0.4)	
Exam resits/supply/reappear number?	0	383 (83.4)	135 (29.4)	248 (54)	9.61 (0.02*)
	1	51 (11.1)	23 (5)	28 (6.1)	
	2	15 (3.3)	10 (2.2)	5 (1)	
	>2	10 (2.2)	6 (1.3)	4 (0.8)	
Do you face any time management problems during your study?	Yes	330 (71.9)	138 (30)	192 (41.8)	7.62 (0.00*)
	No	129 (28.1)	36 (7.8)	93 (20.2)	

The most important factor that causes dropout thoughts

As shown in Table 5, 50 (11%) students contemplating dropout identified inadequate academic achievement as the primary factor contributing to these concerns. Forty-two (9.2%) students identified a non-supportive academic atmosphere as a significant factor contributing to dropout rates.

**Table 5:** The most important factor that causes the thought of dropping out from medical college.

Variables	Frequency (%)	Thoughts of dropping out from medical school (frequency)	
		Yes	No
Poor academic performance	156 (34)	50 (11)	106 (23)
Non-supportive academic environment	116 (25.3)	42 (9.2)	74 (16.1)
Financial problems	5 (1.1)	2 (0.4)	3 (0.6)
Peer pressure	35 (7.6)	18 (3.9)	17 (3.7)
Unsatisfactory methodology of teaching	33 (7.2)	18 (3.9)	15 (3.3)
Personal problems	72 (15.7)	27 (5.9)	45 (9.8)
Family pressure	12 (2.6)	3 (0.6)	9 (1.9)
Other	30 (6.5)	14 (3)	16 (3.5)

## Discussion

This study sought to evaluate the frequency of dropout ideation and its influencing factors among medical students at LMDC. This study is, to our knowledge, the first to exclusively investigate dropout and influencing factors among medical students in Lahore. The results are noteworthy, with 37.9% of students at LMDC indicating thoughts of withdrawal, a much elevated rate compared to the 25.2% observed in previous research [7,14]. Furthermore, 28.7% of students in our survey seriously contemplated withdrawing from medical school, in contrast to the 11% reported in a prior study [5].

Prior studies indicate that the initial year, especially the first year, is the predominant period for students to form an intent to drop out, corroborating our results, wherein 56.3% of first-year students expressed thoughts of withdrawal [7,14,15]. A study from Portugal presented a contrary perspective, indicating that the second year is the most prevalent for dropout considerations [16].

No significant association was identified between demographic characteristics and intentions to drop out. Among the 174 (37.8%) students who contemplated dropping out, 99 (21.5%) were women, which contradicts previous research indicating a higher prevalence of such ideas among men [17]. Concerning marital status, merely 17 (3.7%) students were married, hence complicating the establishment of any substantial association between marital status and thoughts of dropping out. Furthermore, among the students, 292 (63.6%) had no family members who were doctors, while the remainder had medical parents or siblings; nonetheless, this factor showed no statistically significant association with thoughts of dropping out which is in accordance with previous studies [18-20].

A majority of students (83, 47.7%) indicated contemplating withdrawal alone on exceptionally stressful days, corroborating results from a study in the Netherlands [19]. There were 50 (28.7%) students who seriously considered withdrawing which was lower than the rates reported by Rogers et al. [21]. The findings indicate that multiple personal, psychological, and academic aspects are associated with the occurrence of severe dropout ideation. Twenty-two (4.8%) students contemplating dropout experienced no financial difficulties, contradicting a study that indicated an association between debt and significant dropout ideation [7]. Eighty-one (17.6%) students contemplating dropout encountered a detrimental incident that adversely affected their academic performance recently, such as a significant impact of personal sickness or the illness of a close family member (p=0.03). This tendency was similarly observed in prior research [17]. One hundred and six (23%) students expressed dissatisfaction with the teaching methodology which has a significant association with satisfaction regarding teaching methodology (p=0.01). This finding is similar to previous studies which show teaching methodology strongly affects dropout thoughts [22,23]. Among individuals not residing at home, 92 (29.4%) expressed dissatisfaction with their accommodation, and 95 (27.8%) reported experiencing homesickness, which may contribute to thoughts of dropping out. Previous research indicates that accommodation-related issues significantly contribute to the likelihood of dropout, particularly among individuals living in dormitories [24].

Among the eight factors examined in this study related to the rise in dropout thoughts, the most prominent appears to be inadequate academic performance among 50 (11%) students, closely followed by an unsupportive academic environment in 42 (9.2%) students who have dropout thoughts. This finding aligns with a prior study [25], which indicated that a decline in academic performance corresponded with an increase in dropout thoughts. The current survey indicates that 126 (27.5%) medical students who have dropout intentions perceive social isolation due to their education. This outcome parallels a previous study [7] indicating that 52% of students perceive their workload as a barrier to social contact. There were 138 (30%) students who encountered issues stemming from poor time management, which is identified as a statistically significant factor contributing to dropout considerations (p=0.00). This represents a similar finding from prior surveys that indicated 41.9% of students were uncertain about and had difficulty in good time management [7,26,27]. Subpar academic achievement is a significant factor contributing to the desire to withdraw from school. Our study indicates that, overall, 110 (23.9%) students having dropout thoughts were dissatisfied with their academic performance (p=0.03). This is substantiated by prior research [28,29]. A notable statistical association exists between dropout considerations and examination reattempts, as 135 students without extra examinations expressed 29.4% intention of withdrawing from the MBBS program, while only 39 (8.5%) individuals with reattempts harbored dropout intentions with statistically significant association (p=0.02). This outcome parallels another study that similarly demonstrates a higher desire to withdraw from medical school corresponding with a rise in exam retakes [18].

The study was conducted in one medical college; therefore, it lacks generalizability. Hence, multicentric studies should be required to find out the main causative factors leading to dropout thoughts and actual dropout among medical students. 

## Conclusions

The incidence of dropout ideation among medical students at LMDC was significantly elevated, with academic difficulties serving as a primary contributing cause. Nevertheless, the proportion of students contemplating exit from medical school was not significantly elevated. To avert the transition from thoughts of dropping out to actual withdrawal, comprehensive academic support programs must be established to detect and assist at-risk students prior to the escalation of these thoughts into withdrawal. A comprehensive examination of the causal elements and their psychological and psychosocial impacts on students may also be undertaken to enhance this study.
